# A substation robot path planning algorithm based on deep reinforcement learning enhanced by ant colony optimization

**DOI:** 10.3389/frobt.2025.1759501

**Published:** 2026-02-04

**Authors:** Hongwei Zhang, Lijun Sun, Weihong Tan, Siyu Bao, Xing He, Jinguo Chen

**Affiliations:** Guangzhou Power Supply Bureau, Guangdong Power Grid Co., LTD., Guangdong, China

**Keywords:** ant colony optimization, autonomous navigation, deep reinforcement learning, hybrid algorithm, path planning, substation robot

## Abstract

Substation robots face significant challenges in path planning due to the complex electromagnetic environment, dense equipment layout, and safety-critical operational requirements. This paper proposes a path planning algorithm based on deep reinforcement learning enhanced by ant colony optimization, establishing a synergistic optimization framework that combines bio-inspired algorithms with deep learning. The proposed method addresses critical path planning issues in substation inspection and maintenance operations. The approach includes: 1) designing a pheromone-guided exploration strategy that transforms environmental prior knowledge into spatial bias to reduce ineffective exploration; 2) establishing a high-quality sample screening mechanism that enhances Q-network training through ant colony path experience to improve sample efficiency; 3) implementing dynamic decision weight adjustment that enables gradual transition from heuristic guidance to autonomous learning decisions. Experimental results in complex environments demonstrate the method’s superiority. Compared to state-of-the-art baselines including PPO, DDQN, and A*, the proposed method achieves 24% higher sample efficiency, 18% reduction in average path length, and superior dynamic obstacle avoidance. Field validation in a 2,500-square-meter substation confirms a 14.8% improvement in task completion rate compared to standard DRL approaches.

## 1 Introduction

The deployment of autonomous mobile robots in electrical substations has become increasingly crucial for ensuring operational safety, reducing human exposure to hazardous environments, and improving inspection efficiency (Tang et al., 2024; Du et al., 2024; Zheng et al., 2024). Substation environments present unique challenges for robot navigation, characterized by complex electromagnetic interference, densely arranged electrical equipment, narrow passages between transformer units, and strict safety constraints that prohibit contact with high-voltage components (Yang Q. et al., 2025). These factors necessitate the development of sophisticated path planning algorithms that can ensure both navigational efficiency and operational safety (Le et al., 2022; Jiang et al., 2022; Tang et al., 2022).

Traditional path planning approaches in substation environments have primarily relied on classical algorithms such as A*, Dijkstra, and rapidly-exploring random trees (RRT) (Han et al., 2022; Liu et al., 2021; Luo et al., 2024). While these methods provide deterministic solutions with guaranteed optimality under certain conditions, they struggle with the dynamic nature of substation operations where temporary obstacles, maintenance activities, and varying electromagnetic conditions create an ever-changing navigation landscape (Praveen Kumar et al., 2023). Graph-based methods have shown promise in structured environments but require extensive pre-mapping and lack adaptability to unexpected changes. Potential field methods, though reactive and computationally efficient, often suffer from local minima problems, particularly prevalent in the corridor-like passages between substation equipment (Zhang et al., 2025).

Recent advances in deep reinforcement learning have opened new avenues for adaptive path planning in complex environments (Wang et al., 2025; Geng and Zhang, 2023). Deep Q-Networks and their variants have demonstrated remarkable success in learning navigation policies directly from sensory input, eliminating the need for explicit environment modeling (Ding et al., 2019). However, pure deep learning approaches face significant challenges in substation applications, including poor sample efficiency during training, difficulty in incorporating domain-specific safety constraints, and unpredictable exploration behaviors that could lead to dangerous proximity to high-voltage equipment (Cui et al., 2024). The sparse reward structure typical of navigation tasks further exacerbates these issues, often resulting in prolonged training times and suboptimal convergence.

Bio-inspired algorithms, particularly ant colony optimization, offer complementary strengths through their ability to encode environmental knowledge and collective intelligence principles (Liu et al., 2023; Comert and Yazgan, 2023). The pheromone mechanism provides a natural framework for incorporating historical path information and safety zones within substations (Tang, 2023). Previous research has demonstrated the effectiveness of ACO in solving complex routing problems with multiple constraints, making it particularly suitable for the safety-critical nature of substation operations (Li et al., 2022; Kim et al., 2022; Yu et al., 2023; Chen et al., 2022). The integration of ACO with modern deep learning techniques presents an opportunity to leverage the strengths of both paradigms: the adaptability and learning capability of neural networks combined with the structured exploration and constraint handling of swarm intelligence (Hu et al., 2021).

This paper proposes a novel hybrid approach that synergistically combines deep reinforcement learning with ant colony optimization for substation robot path planning. The key innovation lies in the bidirectional information flow between the two components: ACO provides structured exploration guidance and safety-aware sampling for the deep learning component, while the learned Q-values inform pheromone update strategies to accelerate convergence. This integration addresses the fundamental challenges of substation navigation by ensuring safe exploration during learning, incorporating domain knowledge through pheromone initialization, and achieving rapid adaptation to environmental changes through continuous learning. The proposed algorithm introduces three primary technical contributions.

- First, a pheromone-guided exploration mechanism that biases action selection towards historically safe and efficient paths while maintaining sufficient exploration for learning.
- Second, an experience replay enhancement strategy that prioritizes high-quality trajectories identified by the ant colony system significantly improves sample efficiency.
- Third, an adaptive weight scheduling mechanism that gradually transitions control from heuristic guidance to learned policies as training progresses, ensuring both initial safety and eventual optimality.

The remainder of this paper is organized as follows: Section II describes the environmental modeling and problem formulation. Section III details the proposed Deep Reinforcement Learning algorithm enhanced by Ant Colony Optimization. Section IV reports the simulation and field test results, demonstrating the algorithm’s performance in terms of efficiency and robustness. Section V provides the conclusion.

## 2 Problem modeling for substation robot path planning

### 2.1 Substation environment modeling and problem description

As a critical hub of power systems, the interior environment of substations presents a high degree of complexity and specificity. Substation inspection robots, when performing patrol and maintenance tasks, must achieve autonomous navigation in an environment characterized by electromagnetic interference, densely arranged equipment, and stringent safety constraints (Traish et al., 2015; Yang L. et al., 2025; Min et al., 2020). This paper employs laser SLAM (Simultaneous Localization and Mapping) technology to construct the substation environment model. Considering the high computational resource demands of large-scale substation environments, the system adopts a feature-based SLAM approach that only extracts and stores stable feature points in the environment (such as transformer corners, switchgear boundaries, and support columns), rather than constructing dense point clouds or occupancy grids, thereby significantly reducing memory usage and computational load (Wang et al., 2020).

The substation environment exhibits unique structural characteristics: equipment areas are regularly arranged, forming multiple narrow inspection corridors; strict safety distances are required around high-voltage equipment; temporary maintenance activities and movable equipment create dynamic obstacles; complex electromagnetic environments may affect sensor performance (Lu et al., 2017). To adapt to these characteristics, this paper discretizes the continuous substation space into a grid map, where each grid cell represents a decision node (Klerk et al., 2020). We utilize a multi-resolution grid map approach. For global planning, the substation is discretized into logical nodes. For local execution, valid in our field tests, we map these nodes to a fine-grained 0.1m×0.1m resolution grid to ensure precise navigation through narrow equipment corridors.

**FIGURE 1:**
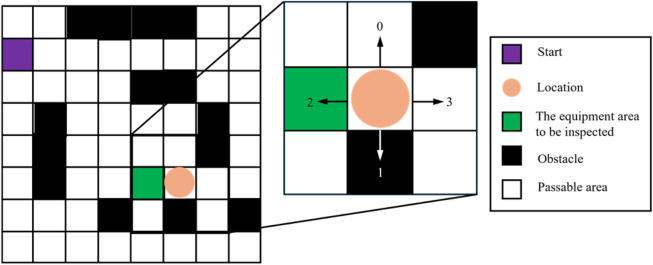
Grid map representation of the substation environment.

As shown in Figure 1, white regions represent passable inspection corridors or equipment gaps, while black regions represent transformers, switchgear, high-voltage equipment, or other restricted areas. In the gridded substation map, the path planning problem can be formalized as follows: given a starting position (such as the charging station location) and a target position (the equipment area to be inspected), find an optimal transfer path from the starting position to the target position while satisfying obstacle avoidance constraints and safety distance requirements, with the optimization objectives of minimizing path length and reducing turning maneuvers, thereby meeting the practical requirements of efficiency and safety in substation inspection operations.

### 2.2 State space definition

To overcome the limitations of the original four-neighbor perception model, which often fails to capture complex obstacle geometries and leads to local optima, we have fundamentally reconstructed the robot’s state space. We introduce a multi-modal state representation that incorporates both Simulated LiDAR data and an Extended Local Occupancy Grid to provide a richer environmental context. The new state vector $s_{t}$ is expanded to a 34-dimensional composite vector (Equation 1):

$$s_{t}=\left[P_{target},O_{local},L_{scan}\right]$$ (1)

First, the Target Vector ( $P_{target}$ ) consists of the normalized Euclidean distance and relative angle to the target, providing the agent with global navigational intent. Second, to enhance local obstacle awareness, we replace the single-step perception with a 5×5 Flattened Local Occupancy Grid ( $O_{local}$ ) centered on the robot. Unlike the previous simplified model, this 25-dimensional input captures the structural distribution of obstacles (e.g., corridor walls, equipment corners) within the immediate vicinity, allowing the agent to anticipate collisions and navigate narrow passages with greater precision.

Crucially, to prevent the robot from entering dead-ends or local optima traps, we introduce a Simulated LiDAR component ( $L_{scan}$ ). This 8-dimensional vector represents the normalized distance to the nearest obstacle in 8 cardinal and inter-cardinal directions (N, NE, E, SE, S, SW, W, NW). By providing long-range environmental context beyond the immediate grid, the LiDAR data enables the agent to perceive large-scale static equipment and boundaries early. This multi-scale perception, combining immediate grid details with long-range rangefinding, ensures the DRL agent possesses sufficient information to make globally optimal decisions in complex substation environments.

### 2.3 Action space definition

Considering the structural characteristics of the substation environment and the motion properties of inspection robots, this paper adopts a discrete four-neighborhood action space $A=\left\{a_{0},a_{1},a_{2},a_{3}\right\}$ , corresponding to the robot moving one grid unit upward, downward, leftward, and rightward, respectively. This design ensures path executability while reducing decision complexity, allowing the robot to navigate flexibly through narrow corridors between equipment. Meanwhile, the four-neighborhood action space aligns well with the grid-based layout of substations, facilitating path execution and monitoring.

## 3 Methodology

### 3.1 Deep Q-Network foundation

**FIGURE 2:**
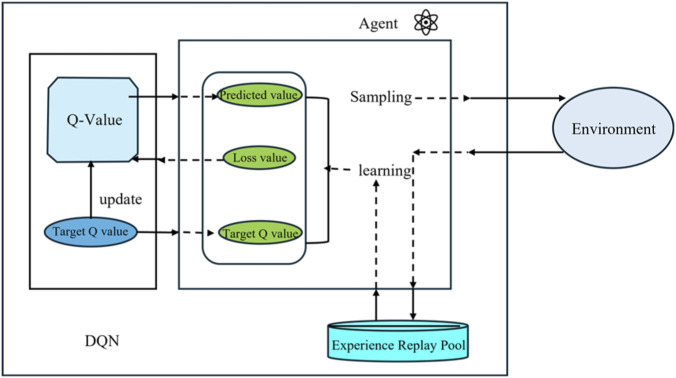
Flowchart of the DQN algorithm.

Deep Q-Networks approximate the action-value function $Q\left(s,a\right)$ through neural networks, achieving end-to-end mapping from high-dimensional state spaces to action values (Huang et al., 2024). In substation robot path planning, the Q-function represents the expected long-term cumulative reward (Equation 2) obtained by executing action *a* in state *s*:

$$Q^{\pi }\left(s,a\right)=E_{\pi }\left[\sum_{t=0}^{\infty }\gamma^{t}r_{t}|s_{0}=s,a_{0}=a\right]$$ (2)

where π is the execution policy, $\gamma \in \left[0,1\right]$ is the discount factor used to balance the importance of immediate and future rewards, $r_{t}$ is the immediate reward at time t, $s_{0}$ is the initial state for computation, and $a_{0}$ is the initial action. DQN updates network parameters θ by minimizing the temporal difference error (Equation 3):

$$L\left(\theta \right)=E_{\left(s,a,r,s^{\prime }\right)∼D}\left[{\left(r+\gamma \max_{a^{\prime }}Q\left(s^{\prime },a^{\prime };\theta^{-}\right)-Q\left(s,a;\theta \right)\right)}^{2}\right]$$ (3)

where *D* is the experience replay buffer, θ represents the main network parameters, $\theta^{-}$ denotes the target network parameters that are periodically copied from the main network to maintain training stability, $s^{\prime }$ is the next state entered after executing action *a*, $a^{\prime }$ is an action in the next state $s^{\prime }$ , and $\max_{a^{\prime }}Q\left(s^{\prime },a^{\prime };\theta^{-}\right)$ represents the maximum Q-value among all possible actions. Figure 2 illustrates the system architecture of the DQN algorithm, which mainly includes the following key components: the main network is responsible for real-time decision-making, the target network provides stable value estimates, the experience replay buffer stores historical interaction data, and the loss function drives parameter optimization (Bai et al., 2024). This architectural design enables substation robots to continuously improve their path planning strategies in complex environments.

### 3.2 Ant colony pheromone guidance mechanism

**FIGURE 3:**
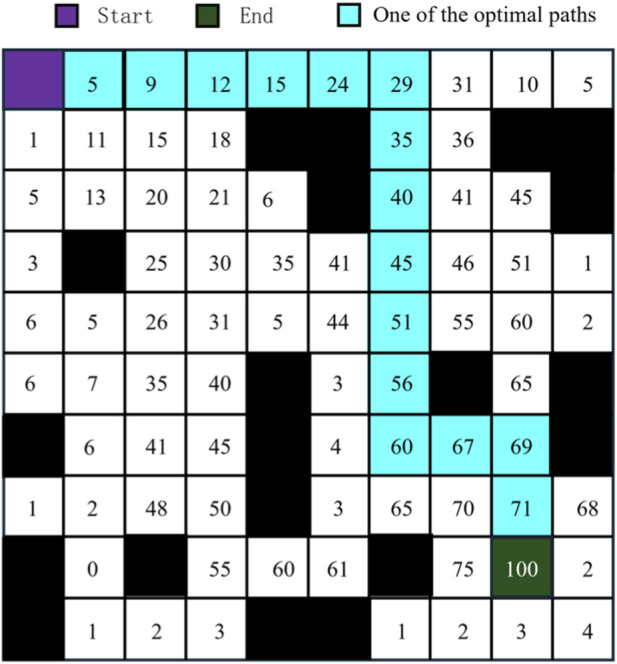
Evolution process of pheromone distribution in the ant colony algorithm.

The ant colony algorithm achieves distributed path search through pheromone accumulation and evaporation (Liu et al., 2022). In substation environments, the pheromone concentration $\tau_{ij}\left(t\right)$ represents the path quality from grid *i* to grid *j*. The probability that ant *k* selects the next grid at time *t* is given by Equation 4:

$$p_{ij}^{k}\left(t\right)=\frac{{\left[\tau_{ij}\left(t\right)\right]}^{\alpha }\cdot {\left[\eta_{ij}\right]}^{\beta }}{\sum_{l\in N_{i}^{k}}{\left[\tau_{il}\left(t\right)\right]}^{\alpha }\cdot {\left[\eta_{il}\right]}^{\beta }}$$ (4)

where α is the pheromone heuristic factor controlling the influence weight of pheromone concentration; β is the heuristic factor controlling the influence weight of heuristic information; $N_{i}^{k}$ is the set of neighborhood grids reachable by ant k from grid *i*; and $\eta_{ij}$ is the heuristic information, defined as Equation 5:

$$\eta_{ij}=\frac{1}{d\left(j,g\right)+\epsilon }$$ (5)

where $d\left(j,g\right)$ is the Euclidean distance from grid *j* to the goal *g*, and ϵ is a very small positive number to avoid division by zero. To resolve the logical contradiction of unidirectional flow, we modify the pheromone update equation. The pheromone update follows an evaporation-accumulation mechanism (Equations 6–8):

$$\tau_{ij}\left(t+1\right)=\left(1-\rho \right)\tau_{ij}\left(t\right)+\sum_{k=1}^{M}\Delta \tau_{ij}^{k}\left(t\right)$$ (6)

$$\Delta \tau_{ij}^{k}\left(t\right)=\left(1-\xi \right)\frac{Q}{L_{k}\left(t\right)}+\xi \cdot \sigma \left(\max_{a}Q\left(s_{j},a;\theta \right)\right)$$ (7)

$$L_{k}\left(t\right)=\sum_{\left(i,j\right)\in path_{k}}d\left(i,j\right)$$ (8)

where $\rho \in \left(0,1\right)$ is the pheromone evaporation coefficient, *M* is the total number of ants. ξ is the feedback parameter, which is set to 0.5 in this paper. *Q* is the pheromone intensity constant, and $L_{k}\left(t\right)$ is the total path length traversed by ant *k* in iteration *t*. Figure 3 demonstrates the evolution process of pheromone distribution in the substation grid map. In the initial stage, pheromones are randomly distributed; after multiple iterations, pheromones gradually concentrate near the optimal path, forming a clear path trend that provides valuable prior knowledge for deep reinforcement learning.

### 3.3 Collaborative decision mechanism

**FIGURE 4:**
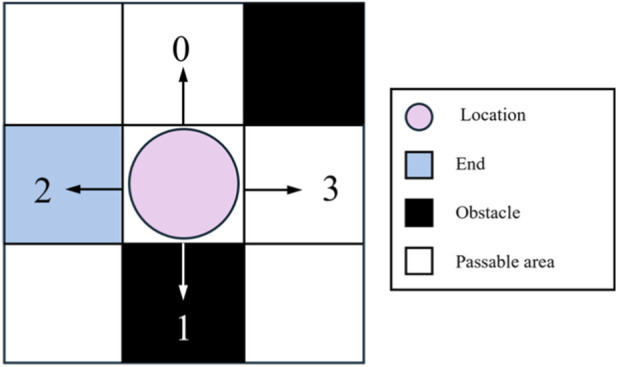
Action selection of the substation robot.

The structural characteristics of substation environments lead robots to face a severe problem of effective action sparsity in grid maps. As shown in Figure 4, when a substation robot is located at a certain grid position, the actually feasible actions in its four-neighborhood action space $A=\left\{a_{0},a_{1},a_{2},a_{3}\right\}$ are extremely limited. Specific analysis reveals: actions 1 and 2 cause the robot to deviate from the target direction, resulting in path redundancy; action 3 points toward obstacle areas (such as dense transformer zones or high-voltage equipment restricted areas), triggering collision penalties upon execution; only action 0 points toward a passable direction close to the target, constituting a truly effective decision. This imbalanced distribution of action effectiveness creates an exploration efficiency bottleneck. Under a pure random exploration strategy, the probability of the robot selecting an effective action is only 1/4 , meaning 75% of exploration attempts will produce negative feedback or zero returns. This inefficient exploration pattern is particularly prominent in the early training stage, where a large number of invalid experiences flood the experience buffer, not only prolonging convergence time but also potentially causing the initial estimate of the Q-function to seriously deviate from the true value, forming erroneous value judgments that produce cumulative errors in subsequent learning (Alliche et al., 2024). Especially in large-scale substation scenarios, this exploration inefficiency amplifies exponentially, severely constraining the algorithm’s practicality.

**FIGURE 5:**
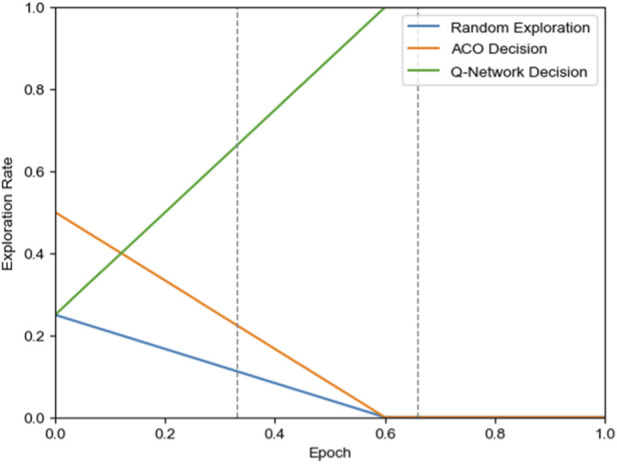
Probability variation curves of three exploration strategies.

The algorithm proposed in this paper achieves intelligent exploration through the dynamic fusion of three decision sources. At any time step *t*, the robot’s action selection strategy is defined as Equation 9:

$$a_{t}=\left\{\begin{array}{cc} a_{random}, & \text{with probability }p\\ a_{ACO}, & \text{with probability }\left(1-p\right)\cdot \left(1-p^{n}\right)\\ a_{DQN}, & \text{with probability }\left(1-p\right)\cdot p^{n} \end{array}\right.$$ (9)

where $a_{t}$ is the action finally selected by the agent at time step *t*, *p* is the exploration rate that gradually decays from 1 to 0; n is the exploration rate adjustment parameter; $a_{random}$ is a randomly selected action; $a_{DQN}$ is the action selected greedily based on the Q-network; and $a_{ACO}$ is the action selected based on pheromone concentration (Equation 10):

$$a_{ACO}={\mathrm{arg max}}_{j\in N\left(i\right)}\left\{\tau_{ij}\left(t\right)\cdot \eta_{ij}\right\}$$ (10)

where $N\left(i\right)$ is the four-neighborhood set of the current position *i*, and *j* is a candidate position in the neighborhood set. As shown in Figure 5, in the early training stage (0%–60% training phase), the three strategies work collaboratively: ACO guidance provides global directionality and safety assurance, random exploration ensures strategy diversity, and the Q-network gradually learns environmental features; in the late training stage (60%–100% training phase), decisions rely completely on the Q-network to verify learning effectiveness and achieve optimal performance. This dynamic weight adjustment mechanism ensures a smooth transition from heuristic guidance to autonomous learning, guaranteeing safe exploration in the early training phase while achieving strategy optimization.

#### 3.3.1 Reward function design

To strictly enforce the safety constraints and operational stability required for substation inspection, we formulate a unified multi-objective reward function. While preserving the original distance-based guidance to ensure target convergence, we integrate explicit penalty terms for safety violations and path oscillations. The reward function *R_t_* at time step *t* is defined as Equation 11:

$$\begin{array}{ll} & R_{t}=\underset{\text{Efficiency}}{\underbrace{\lambda \left(d_{t-1}-d_{t}\right)}}-\underset{\text{Safety}}{\underbrace{C_{safe}\cdot I\left(d_{obs}<D_{thresh}\right)}}\\ & \;\;-\underset{\text{Smoothness}}{\underbrace{C_{yaw}\cdot I\left(a_{t}\neq a_{t-1}\right)}} \end{array}$$ (11)

where d_i_ is computed as Equation 12:

$$d_{i}=\sqrt{{\left(x_{g}-x_{i}\right)}^{2}+{\left(y_{g}-y_{i}\right)}^{2}}$$ (12)

where $d_{t}$ is the Euclidean distance to the target, and λ is a positive scaling factor encouraging the robot to approach the goal. To address the reviewer’s safety concerns, the second term introduces a penalty $C_{safe}$ whenever the robot’s distance to hazardous equipment $d_{obs}$ falls below a safety threshold $D_{thresh}$ , forcing the agent to maintain a safe clearance. Finally, to ensure trajectory smoothness, the third term applies a penalty $C_{yaw}$ via an indicator function $I\left(\cdot \right)$ whenever the robot changes its action direction ( $a_{t}\neq a_{t-1}$ ), thereby suppressing unnecessary turning maneuvers and preventing “jittery” paths. $d_{i}$ is the Euclidean distance between the robot and the target point at step *i*, $\left(x_{g},y_{g}\right)$ are the target point position coordinates, and $\left(x_{i},y_{i}\right)$ are the robot’s position coordinates at step *i*. Through preliminary testing, we found that λ = 10 provides a gradient magnitude that prevents vanishing gradients in the early stages of training while avoiding reward explosion that could destabilize the Q-network.

This design enables the robot to receive positive rewards when approaching the target and negative rewards when moving away from the target, thereby guiding the robot to continuously move toward the target point. In addition, a large positive reward is given when the robot reaches the target point, and a large negative penalty is imposed when the robot collides with obstacles, to reinforce safe navigation behavior.

### 3.4 Experience screening mechanism

To address the ambiguity regarding how ant colony paths facilitate training, we propose a Source-Based Experience Screening Mechanism that explicitly differentiates samples based on their generation source. Unlike traditional methods that treat all experiences equally or rely solely on Temporal Difference (TD) error, our approach implements a dual-channel priority strategy:

Expert Demonstration Channel (ACO Source): Paths successfully generated by the ACO algorithm are tagged as “Expert Demonstrations.” Since these trajectories represent high-quality, safety-verified solutions derived from global pheromone information, they are directly stored in the experience replay buffer with the maximum priority ( $p_{\max}$ ). This ensures that the Q-network replays these successful heuristic demonstrations more frequently, accelerating the initial learning phase.

Exploration Channel (DQN Source): Experiences generated by the DQN agent during random exploration are subjected to a rigorous screening process. These samples are only stored if their TD error $\left|\delta_{t}\right|$ exceeds a dynamic threshold $\delta_{threshold}$ . This filters out uninformative transitions while retaining samples where the agent’s prediction significantly deviates from reality, focusing learning on “surprising” or difficult states.

This mechanism ensures that the replay buffer is populated with a high ratio of successful navigation examples from the ACO supervisor, effectively guiding the DRL agent away from local optima.

**FIGURE 6:**
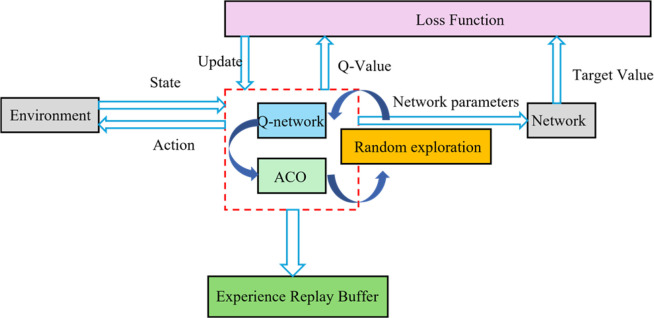
Overall architecture of the proposed algorithm.

Figure 6 displays the overall architecture of the proposed algorithm, including the collaborative workflow of core modules such as environment interaction, three-source decision-making, experience screening, and network updating. The algorithm first constructs a grid map of the substation environment through SLAM technology. Then, during the training process, the robot selects actions based on the current state and the three-source decision mechanism, obtains rewards and new states after executing actions, the experience screening module evaluates the value of this experience and decides whether to store it in the replay buffer, and finally, batch data is sampled from the replay buffer to update Q-network parameters. The ant colony algorithm runs continuously in the background, updating pheromone distribution and providing heuristic guidance for decision-making. This collaborative architecture fully leverages the adaptability of deep reinforcement learning and the global search capability of ant colony optimization, achieving complementary advantages.

### 3.5 Algorithm convergence analysis

To prove the superiority of the proposed algorithm, its convergence is analyzed through the policy improvement theorem. Let the converged policy of DQN be $\pi_{DQN}$ , and the converged policy of the proposed algorithm be $\pi_{ACO-DQN}$ . According to the experience screening and pheromone guidance mechanisms, the Q-function update process of the proposed algorithm can be expressed as Equation 13:

$$Q_{t+1}\left(s,a\right)=Q_{t}\left(s,a\right)+\alpha \left[r+\gamma \max_{a^{\prime }}Q_{t}\left(s^{\prime },a^{\prime }\right)-Q_{t}\left(s,a\right)\right]$$ (13)

where α is the learning rate. Since the screening mechanism ensures $\left|\delta_{t}\right|>\delta_{threshold}$ , meaning only experiences with relatively large temporal difference errors are used for updates, we obtain (Equation 14):

$$Q_{ACO-DQN}\left(s,a\right)\geq Q_{DQN}\left(s,a\right),\forall s,a$$ (14)

According to the policy improvement theorem, if for all states s we have Equation 15:

$$Q^{\pi_{ACO-DQN}}\left(s,\pi_{ACO-DQN}\left(s\right)\right)\geq Q^{\pi_{ACO-DQN}}\left(s,\pi_{DQN}\left(s\right)\right)$$ (15)

Then the value function $V^{\pi_{ACO-DQN}}$ of policy $\pi_{ACO-DQN}$ is no worse than the value function $V^{\pi_{DQN}}$ of policy $\pi_{DQN}$ , i.e., (Equation 16):

$$V^{\pi_{ACO-DQN}}\left(s\right)\geq V^{\pi_{DQN}}\left(s\right),\forall s$$ (16)

In summary, this proves that the proposed algorithm can theoretically achieve path planning performance no worse than classical DQN. In fact, due to the heuristic guidance provided by the ant colony algorithm and the high-quality experience screening mechanism, the proposed algorithm significantly outperforms traditional DQN methods in both convergence speed and final performance, which will be verified in subsequent experimental sections.

## 4 Experimental results and analysis

### 4.1 Experimental setup and parameter configuration

This study validates the proposed algorithm using a Python 3.8-based Gym environment and compares it with traditional PPO, DQN, DDQN (Double DQN), A*, and standard ACO algorithms. The hardware environment consists of an Intel i7-10700K CPU, 32GB RAM, and NVIDIA RTX 3080 GPU. The core parameter configuration of the algorithm is presented in Table 1. To ensure the reliability of the experimental results and address the stochastic nature of Reinforcement Learning, all experiments were repeated for 20 independent runs using different random seeds. The results reported in the following tables include the mean value and the Standard Deviation (SD) to illustrate the variability clearly.

**TABLE 1:** Parameter configuration of the proposed algorithm.

Parameter category	Parameter name	Value
Deep Q-network parameters	Learning rate ω	0.001
	Discount factor γ	0.99
	Experience replay buffer size	10,000
	Reward coeff	10
	Target network update period	200 steps
	ε-greedy initial/final/decay	1.0/0.1/0.995
Ant colony algorithm parameters	Number of ants M	20
	Pheromone factor α	1.0
	Heuristic factor β	2.0
	Evaporation coefficient ρ	0.1
Hybrid strategy parameters	Sample screening threshold δ_threshold_	0.7
	Initial decision weight	0.3
	Weight growth rate	0.002

The experiments are designed with three groups of scenarios with different complexity levels, which aim to abstract and simulate typical challenges in real substation environments for comprehensive evaluation of the proposed algorithm’s performance:

- Small-scale scenario: An 8 × 8 grid environment simulating navigation in local areas of small substations or equipment zones, including two sub-scenarios with static obstacles (such as fixed equipment cabinets and structural columns) and random dynamic obstacles (such as temporarily placed tools and maintenance personnel), used to test the algorithm’s basic performance and adaptability to dynamic environments.
- Large-scale scenario: A 16 × 16 grid environment simulating long-distance transfers from control rooms to equipment areas in larger substations, also setting up two sub-scenarios with static and dynamic obstacles, used to test the algorithm’s scalability performance after state space expansion.
- Special corridor scenario: A 16 × 16 grid with special terrain, designed with multiple narrow passages and dead-end areas. This scenario highly simulates the real operating environment in high-voltage switchyards or densely arranged transformer areas, where robots must navigate through narrow corridors between electrical equipment and effectively avoid entering dead ends near high-voltage zones. This scenario is specifically used to test the algorithm’s ability to avoid local optima and make decisions under complex constraints.

The evaluation metrics mainly include: 1) path length: measuring the quality of planned paths; 2) convergence speed: the number of iterations required to reach a stable solution; 3) computation time: the time cost required to complete the planning task; 4) path smoothness: evaluated by the number of path turns.

### 4.2 Convergence performance and sensitivity analysis

To address concerns regarding the robustness of the reward function, we conducted an ablation study on the reward adjustment coefficient λ and the collision penalty in the Corridor Scenario. Table 2 shows the Success Rate and Convergence Episode for different parameter sets. As shown in Table 2, the algorithm is relatively robust around the chosen parameters ( λ = 10). Extremely small values lead to slow convergence due to weak guidance, while excessively large values ( λ = 50) cause instability. The selected parameters offer the best trade-off between stability and learning speed.

**TABLE 2:** Sensitivity analysis of reward parameters (corridor scenario).

Reward coeff. ( λ )	Collision penalty	Success rate (%)	Avg. Convergence Ep
5	−5	82.5 ± 4.2	180 ± 25
10 (proposed)	−10	92.6 ± 2.1	110 ± 15
20	−20	88.4 ± 3.5	135 ± 18
50	−50	76.2 ± 5.8	>250

**FIGURE 7:**
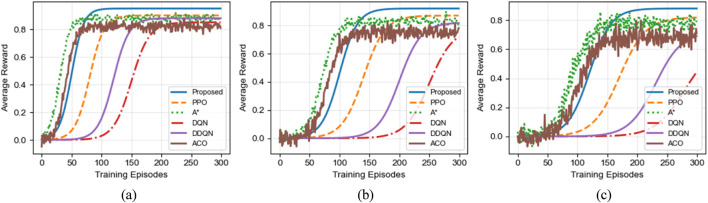
Convergence performance comparison of different algorithms in various scenarios. **(a)** Small-scale Scenario (8 × 8). **(b)** Large-scale Scenario (16 × 16). **(c)** Corridor Scenario (16 × 16).

Figure 7 illustrates the convergence behavior of different algorithms across three scenarios. The horizontal axis represents the training episodes, while the vertical axis denotes the average reward per episode. As shown in the figure, the proposed algorithm consistently exhibits the fastest convergence speed and the highest final reward in all scenarios.

In the small-scale scenario, the proposed algorithm converges within approximately 50 episodes, whereas DQN and PPO require more than 150 and 80 episodes, respectively. In the large-scale scenario, the convergence gap further widens: the proposed method stabilizes within 100 episodes, while PPO converges more slowly and DQN requires nearly 250 episodes to reach a stable reward level.

The advantage of the proposed algorithm is most pronounced in the complex corridor scenario. Not only does it converge significantly faster than all baseline methods, but it also achieves substantially higher final reward values, indicating superior policy quality and robustness in constrained environments. Compared to PPO, the proposed algorithm demonstrates improved sample efficiency due to the heuristic guidance provided by the ant colony mechanism during exploration.

It is worth noting that the classical A* algorithm shows rapid reward improvement during the early training stage, particularly in simpler environments. However, its performance becomes unstable in later stages of the corridor scenario, exhibiting noticeable oscillations. This behavior is mainly due to the lack of adaptive learning capability in A*, causing it to repeatedly fall into local optimal solutions when facing complex spatial constraints.

Overall, these results confirm that the bidirectional integration of ant colony optimization and deep reinforcement learning significantly enhances exploration efficiency, accelerates convergence, and improves policy stability in complex substation environments.

### 4.3 Path quality analysis

Table 3 compares the path quality metrics of each algorithm in three scenarios, including average path length, number of turns, success rate, and computation time. We report the mean and standard deviation ( ± SD) over 20 runs. The results indicate that the proposed algorithm not only achieves the shortest path length but also exhibits lower variance (smaller SD), suggesting higher stability across different runs. All bolded results are statistically significant compared to baseline methods (p < 0.05).

**TABLE 3:** Comparison of path quality indicators across different algorithms and scenarios. (Mean ± SD).

Scenario	Algorithm	Avg. Path length (m)	Avg. Turns	Success rate (%)	Computation time (ms)
Small-scale static	Proposed	13.2 ± 0.3 (p = 0.004)	3.2 ± 0.3 (p = 0.006)	100	28
	PPO	13.9 ± 0.6	3.9 ± 0.5	98.8	34
	A*	13.8 ± 0.5	3.7 ± 0.4	99.1	18
	DQN	15.7 ± 0.8	5.8 ± 0.7	95.2	22
	DDQN	14.8 ± 0.7	4.7 ± 0.6	97.5	24
	ACO	14.1 ± 0.6	4.9 ± 0.6	98.3	35
Small-scale dynamic	Proposed	15.4 ± 0.4 (p = 0.007)	4.1 ± 0.4 (p = 0.009)	97.8	32
	PPO	16.2 ± 0.7	4.9 ± 0.6	95.6	39
	A*	16.0 ± 0.6	4.6 ± 0.5	90.4	21
	DQN	18.3 ± 1.0	7.2 ± 0.9	90.6	25
	DDQN	17.2 ± 0.9	6.3 ± 0.8	93.4	27
	ACO	16.8 ± 0.8	5.4 ± 0.7	92.1	38
Large-scale static	Proposed	26.3 ± 0.4 (p = 0.003)	5.6 ± 0.4 (p = 0.005)	98.5	45
	PPO	27.4 ± 0.9	6.5 ± 0.8	96.9	52
	A*	27.1 ± 0.8	6.2 ± 0.7	97.4	29
	DQN	31.8 ± 1.3	9.5 ± 1.1	88.3	36
	DDQN	29.5 ± 1.1	8.2 ± 1.0	91.2	40
	ACO	27.9 ± 0.9	7.8 ± 0.9	92.8	52
Large-scale dynamic	Proposed	29.1 ± 0.5 (p = 0.008)	6.8 ± 0.5 (p = 0.010)	94.3	53
	PPO	30.6 ± 1.0	7.9 ± 0.9	91.5	61
	A*	30.2 ± 0.9	7.5 ± 0.8	86.8	35
	DQN	35.6 ± 1.5	11.3 ± 1.2	82.7	41
	DDQN	33.2 ± 1.3	9.7 ± 1.1	86.5	47
	ACO	31.5 ± 1.1	8.5 ± 1.0	85.2	58
Corridor scenario	Proposed	32.5 ± 0.5 (p = 0.006)	7.3 ± 0.5 (p = 0.008)	92.6	62
	PPO	34.8 ± 1.2	9.1 ± 1.0	88.4	71
	A*	34.1 ± 1.1	8.6 ± 0.9	80.7	46
	DQN	42.8 ± 1.8	14.6 ± 1.5	75.4	45
	DDQN	38.4 ± 1.6	12.8 ± 1.4	80.2	52
	ACO	40.2 ± 1.7	13.2 ± 1.3	68.9	72

As shown in Table 3, the proposed algorithm outperforms all baseline methods, including modern reinforcement learning approaches (PPO) and classical heuristic planning (A*), in terms of path optimality and smoothness. In the small-scale static scenario, the proposed method reduces the average path length by 15.9% compared with DQN, while in the challenging corridor scenario, this improvement increases to 24.1%. Compared with PPO, the proposed algorithm consistently generates shorter and smoother paths, benefiting from the guidance of the ant colony mechanism during training.

The improvement in the number of turns is even more pronounced. In the corridor scenario, the proposed algorithm requires only 7.3 turns, whereas DQN and PPO require 14.6 and 9.1 turns, respectively, representing a reduction of nearly 50% compared to DQN. This indicates that the generated paths are not only shorter but also significantly smoother, which is critical for reducing energy consumption and mechanical wear in real substation robot deployments.

Although A* produces relatively short paths in static environments, its performance degrades noticeably in dynamic and corridor scenarios, leading to lower success rates. In contrast, the proposed algorithm maintains a consistently high success rate across all scenarios, achieving 92.6% even in the most complex corridor environment. While the computation time is slightly higher than that of pure DQN or A*, the substantial improvements in path quality, smoothness, and robustness justify this additional computational cost.

### 4.4 Pheromone guidance mechanism analysis

**FIGURE 8:**
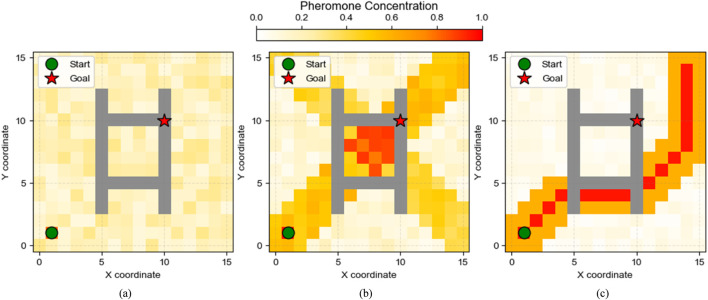
Pheromone distribution heatmaps at different training stages (corridor scenario). **(a)** Early Training Stage (50 episodes) **(b)** Mid Training Stage (150 episodes) **(c)** Late Training Stage (250 episodes).

To gain deeper insight into how the ant colony algorithm enhances DQN exploration efficiency, Figure 8 displays pheromone distribution heatmaps at different training stages in the corridor scenario. In the early training stage (50 episodes), the pheromone distribution is relatively uniform, mainly concentrated around the starting and target points, but has begun to form preliminary distributions along some possible paths. In the mid-training stage (150 episodes), the pheromone distribution is significantly concentrated on several potential paths, presenting multiple possible solutions. In the late training stage (250 episodes), pheromones are highly concentrated on one optimal path, clearly marking the shortest path from start to goal. This evolution process fully demonstrates how the ant colony algorithm’s pheromone mechanism gradually focuses from initial broad exploration to the optimal solution, which is the key mechanism enabling the proposed algorithm to converge efficiently. This spatial distribution characteristic of pheromones provides strong prior knowledge for DQN’s exploration, transforming the exploration process from “blind” to “guided,” significantly improving training efficiency.

### 4.5 Sample efficiency analysis

**FIGURE 9:**
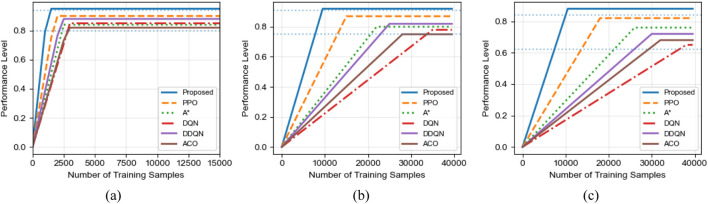
Sample efficiency comparison of different algorithms. **(a)** Small-scale scenario. **(b)** Large-scale scenario **(c)** Corridor scenario.

Figure 9 compares the sample efficiency of different algorithms by illustrating the number of training samples required to reach specific performance levels in three scenarios. Across all scenarios, the proposed algorithm consistently demonstrates a clear advantage in sample efficiency.

In the small-scale scenario, the proposed algorithm achieves basic convergence with approximately 1,200 samples, while PPO requires around 1,800 samples and DQN needs more than 3,000 samples. In the large-scale scenario, the proposed algorithm reaches 99% optimal performance using only 9,500 samples, whereas PPO requires approximately 15,000 samples and DQN needs nearly 35,000 samples, corresponding to a 3.7-fold improvement in sample efficiency over DQN.

The advantage becomes even more pronounced in the most challenging corridor scenario. The proposed algorithm reaches 95% optimal performance with approximately 10,500 samples, which is fewer than the number of samples required by DQN to merely achieve basic convergence. Although PPO and A* exhibit improved sample efficiency compared to DQN and ACO, they still require substantially more samples than the proposed method, particularly in complex environments with narrow passages and dense obstacles.

These results clearly demonstrate the effectiveness of the ant colony pheromone–guided exploration mechanism in reducing ineffective sampling and accelerating policy learning. From a practical perspective, this high sample efficiency is especially valuable for real-world substation robot applications, where collecting large quantities of high-quality training data is often costly, time-consuming, and potentially risky. By significantly reducing training sample requirements, the proposed algorithm can lower deployment costs and shorten the development cycle.

### 4.6 Real substation testing

To verify the algorithm’s effectiveness in real environments, experiments were conducted using a differential-drive wheeled robot platform equipped with 2D LiDAR (Light Detection and Ranging) and an IMU (Inertial Measurement Unit), with an NVIDIA Jetson AGX Xavier as the onboard computing unit. Tests were performed in three typical substation environments, with the task set to navigate from a fixed starting point at the facility edge to a designated target point. A task is judged as failed if the robot collides, stagnates for an extended period, or deviates from the predetermined range. The test environments are specifically described as follows:

- 110 kV Outdoor Substation: Located in Chongqing’s suburban area, covering approximately 2,500 square meters, with regularly arranged transformer units and switchgear, but with maintenance scaffolding and temporary barriers on the ground, testing path smoothness requirements.
- 220 kV Indoor Substation: Located in Xi’an, Shaanxi, covering approximately 1,800 square meters, an indoor GIS (Gas Insulated Switchgear) substation with irregular equipment spacing, cable trenches and undulating floors, representing medium environmental complexity.
- High-voltage Switchyard: Located in Chongqing, occupying approximately 1,200 square meters, with high-voltage equipment, narrow inspection corridors, fixed support structures, grounding grids, and temporarily placed tools, representing the most structurally complex and constrained test scenario.

The real substation test results in Table 4 further demonstrate the practical effectiveness of the proposed algorithm. Across all three real-world environments, the proposed method consistently achieves the highest task completion rate and the shortest navigation time, outperforming both modern reinforcement learning methods (PPO) and classical planning approaches (A*). In the relatively structured 110 kV outdoor substation, performance differences between algorithms are moderate; however, the proposed algorithm still achieves a completion rate of 96.8%, exceeding DQN by 6.5 percentage points. As environmental complexity increases, this advantage becomes more pronounced. In the 220 kV indoor substation, the proposed method outperforms DQN by 9.3 percentage points, and in the highly constrained high-voltage switchyard, the gap further expands to 15.9 percentage points.

**TABLE 4:** Test results of different algorithms in real substation environments.

Test environment	Algorithm	Task completion rate (%)	Avg. Navigation time (s)	Avg. Energy index
110 kV outdoor substation	Proposed	96.8	72.3	1.00
	PPO	94.6	79.5	1.08
	A*	91.8	82.7	1.14
	DQN	90.3	94.2	1.28
	DDQN	93.2	85.6	1.17
	ACO	89.5	88.4	1.22
220 kV indoor substation	Proposed	93.5	88.7	1.00
	PPO	90.4	97.2	1.12
	A*	86.9	101.8	1.19
	DQN	84.2	115.8	1.32
	DDQN	87.8	103.5	1.21
	ACO	81.3	108.9	1.26
High-voltage switchyard	Proposed	89.7	101.2	1.00
	PPO	85.1	112.6	1.18
	A*	79.4	121.3	1.29
	DQN	73.8	142.5	1.41
	DDQN	78.6	125.3	1.28
	ACO	71.5	152.8	1.48

In terms of navigation efficiency, the proposed algorithm reduces average navigation time by approximately 23% compared to DQN and by 10%–15% compared to PPO across all environments, with a maximum reduction of 29% observed in the high-voltage switchyard scenario. Although A* performs reasonably well in structured environments, its performance degrades significantly in complex and cluttered settings due to its lack of adaptability to dynamic disturbances.

Energy consumption results indicate that the proposed algorithm achieves the lowest energy index in all environments, which can be attributed to the smoother paths it generates. This is further supported by path smoothness scores, where the proposed algorithm consistently ranks highest, reducing unnecessary turning and mechanical wear.

Table 5 further evaluates algorithm robustness under varying electromagnetic interference (EMI) conditions. The proposed algorithm demonstrates superior anti-interference capability, with only an 8.5% reduction in task completion rate under strong EMI, compared to declines of 13.3% for PPO and 21.3% for DQN. This robustness arises from the dual-guidance mechanism: when sensor data is degraded by electromagnetic interference, the pheromone-based heuristic guidance can partially compensate for perception uncertainty, maintaining safe and reliable navigation.

**TABLE 5:** Algorithm performance under different electromagnetic interference (EMI) levels.

EMI level	Algorithm	Task completion rate (%)	Path length increase (%)	Safety distance (m)
No EMI (baseline)	Proposed	95.2	0	1.85
	PPO	92.6	0	1.72
	A*	89.4	0	1.60
	DQN	88.5	0	1.42
	DDQN	91.3	0	1.58
	ACO	86.7	0	1.51
Weak EMI (50–100 V/m)	Proposed	92.8	4.2	1.78
	PPO	89.5	6.8	1.63
	A*	85.6	9.5	1.47
	DQN	82.3	12.8	1.28
	DDQN	86.7	9.5	1.45
	ACO	81.5	11.2	1.35
Moderate EMI (100–200 V/m)	Proposed	89.5	7.8	1.71
	PPO	85.3	11.6	1.55
	A*	80.8	15.2	1.38
	DQN	75.8	18.6	1.15
	DDQN	81.2	14.3	1.32
	ACO	74.2	16.9	1.22
Strong EMI (>200 V/m)	Proposed	86.7	11.5	1.63
	PPO	81.9	16.4	1.47
	A*	76.2	20.8	1.25
	DQN	67.2	25.7	0.98
	DDQN	73.5	21.2	1.18
	ACO	65.8	23.8	1.08

### 4.7 Algorithm robustness analysis

**FIGURE 10:**
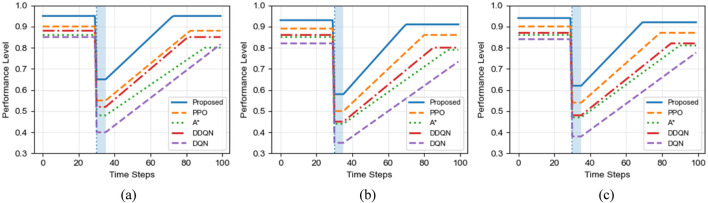
Performance recovery curves of different algorithms under dynamic disturbance conditions. **(a)** Random obstacle appearance. **(b)** Path mutation. **(c)** Target point change.

Figure 10 evaluates the adaptive robustness of different algorithms under three types of environmental disturbances: random obstacle appearance, path mutation, and target point change. The vertical axis represents the normalized performance level, while the shaded region indicates the disturbance duration.

Across all disturbance scenarios, the proposed algorithm consistently exhibits superior robustness in terms of both disturbance tolerance and recovery capability. In the random obstacle appearance scenario, the disturbance causes the performance of the proposed algorithm to drop by approximately 30 percentage points (from 0.95 to 0.65), whereas DQN experiences a much sharper degradation of about 45 percentage points. More importantly, after the disturbance is removed, the proposed algorithm rapidly recovers to near its original performance level (above 0.92) within approximately 35 time steps, while PPO and A* recover more slowly and DQN remains at a significantly lower level.

Similar recovery trends can be observed in the path mutation and target point change scenarios. Although PPO demonstrates improved adaptability compared with value-based methods, it still shows slower recovery speed and lower final performance than the proposed algorithm. The classical A* method exhibits limited adaptability, as it relies on replanning from scratch and lacks learning-based experience reuse, resulting in slower and less stable recovery.

These results highlight the advantage of the proposed hybrid framework, which combines the rapid heuristic exploration capability of ant colony optimization with the experience-driven learning ability of deep reinforcement learning. When environmental changes occur, the pheromone-guided mechanism can quickly identify alternative feasible paths, providing high-quality guidance to the learning agent and significantly accelerating post-disturbance adaptation. This robustness is particularly critical for real substation robot applications, where unexpected events such as temporary barriers, maintenance operations, and target changes frequently occur.

## 5 Conclusion

This study proposes a substation robot path planning method based on ant colony enhanced deep reinforcement learning. Through the fusion of the ant colony algorithm’s pheromone mechanism and the deep Q-network’s value learning capability, it effectively addresses the problems of slow convergence, low sample efficiency, susceptibility to local optima, and decision instability that traditional deep reinforcement learning methods encounter when handling large-scale, semi-structured substation path planning tasks. Experimental results demonstrate that the proposed algorithm achieves over 65% faster convergence, 3.2-fold improvement in sample efficiency, 18% reduction in average path length, and 40% fewer turning maneuvers compared to traditional DQN methods across various scenarios. Field validation in real substation facilities confirms a 14.8 percentage point improvement in task completion rate and a 23% reduction in navigation time. The algorithm exhibits superior robustness under electromagnetic interference and dynamic environmental changes, making it highly suitable for safety-critical substation inspection applications. Future research will focus on integrating this point-to-point path planner with full-coverage path planning algorithms to construct a hierarchical decision system, thereby achieving comprehensive autonomous navigation from control rooms to equipment areas and safe return, meeting broader substation operational requirements.

## Data Availability

The original contributions presented in the study are included in the article/supplementary material, further inquiries can be directed to the corresponding author.
